# Gene duplication and the evolution of moonlighting proteins

**DOI:** 10.3389/fgene.2015.00227

**Published:** 2015-07-07

**Authors:** Adriana Espinosa-Cantú, Diana Ascencio, Francisco Barona-Gómez, Alexander DeLuna

**Affiliations:** Laboratorio Nacional de Genómica para la Biodiversidad (Langebio), Centro de Investigación y de Estudios Avanzados del Instituto Politécnico Nacional (CINVESTAV), Irapuato, Mexico

**Keywords:** moonlighting proteins, gene duplication and evolution, genetic redundancy, subfunctionalization, neofunctionalization, dosage balance, functional trade-offs, paralog responsiveness

## Abstract

Gene duplication is a recurring phenomenon in genome evolution and a major driving force in the gain of biological functions. Here, we examine the role of gene duplication in the origin and maintenance of moonlighting proteins, with special focus on functional redundancy and innovation, molecular tradeoffs, and genetic robustness. An overview of specific examples-mainly from yeast-suggests a widespread conservation of moonlighting behavior in duplicate genes after long evolutionary times. Dosage amplification and incomplete subfunctionalization appear to be prevalent in the maintenance of multifunctionality. We discuss the role of gene-expression divergence and paralog responsiveness in moonlighting proteins with overlapping biochemical properties. Future studies analyzing multifunctional genes in a more systematic and comprehensive manner will not only enable a better understanding of how this emerging class of protein behavior originates and is maintained, but also provide new insights on the mechanisms of evolution by gene duplication.

## Current Views on the Evolution of Moonlighting Proteins

Moonlighting proteins are thought to originate by evolutionary “tinkering” or “co-option” whereby functional novelties arise neutrally in pre-existent scaffolds and eventually result in biochemical advantages (Jacob, 1977; Gancedo and Flores, 2008; Flores and Gancedo, 2011). However, this scenario is seemingly in conflict with the fact that mutations in the coding sequences of genes tend to be deleterious (Ng and Henikoff, 2006; Tokuriki and Tawfik, 2009b; Soskine and Tawfik, 2010). Thus, the tradeoff between sequence-space exploration and maintenance of an ancestral activity needs to be overcome in the origin of new moonlighting roles. Some structural properties may alleviate such tradeoffs. For instance, flexible and modular proteins tend to have higher probabilities to accumulate mutations without deleterious effects on their original structure and function (James and Tawfik, 2003). Likewise, the flexibility of disordered regions and loops in proteins may allow the adaptation to different functional interactions without major tradeoffs (Tompa et al., 2005; Tokuriki and Tawfik, 2009b; Flores and Gancedo, 2011; Noda-García and Barona-Gómez, 2013).

Mutations in *cis* or in *trans* may also enhance a new function without an associated evolutionary tradeoff. In other words, mutations leading to novel functions may occur not only within the open reading frame of a gene, but also in its promoter region or in an interacting gene (Copley, 2014). Changes in the regulatory regime may alter the spatial–temporal expression pattern or the effective dosage of molecules, and therefore increase the probability of a physical interaction. For example, a new cellular environment may favor a structural conformation and a protein–protein interaction that leads to the origin of a new molecular role.

Previous statements about the role of gene duplication in the evolution of moonlighting proteins have focused on their loss by specialization of the coexisting functions. Duplication and divergence of a protein with more than one molecular activity may lead to partitioned functions in the duplicates, and result in monofunctional specialists (Huberts and van der Klei, 2010; Copley, 2014). For example, the ancestral moonlighting argininosuccinate lyase/δ-crystallins from chicken was duplicated and further specialized: Today, one duplicate has no enzymatic activity and is expressed abundantly in lenses, while the other copy is an enzymatically active protein that is preferentially expressed in heart and brain (Li et al., 1993). Here, we propose that, in addition to its role in the loss of moonlighting proteins by functional specialization, gene duplication influences the origin and maintenance of these kind of multifunctional molecules, which can take place even in the presence of tradeoffs between coexisting molecular functions.

## Evolution by Gene Duplication

Gene duplication is a major driving force in the evolution of new biological functions and an important mechanism that renders biological systems robust to genetic and environmental perturbations. The general idea is that the presence of two or more gene copies in a genome provides a “back-up” mechanism that allows organisms to be phenotypically stable under a variety of genetic, environmental, or stochastic perturbations (Gu et al., 2003; Wagner, 2005). In what follows, we review models of evolution by gene duplication, after which we discuss their implications in the evolution of moonlighting proteins.

After duplication and divergence, most gene copies are lost and become pseudogenes by the accumulation of loss-of-function mutations. Different models have been proposed to describe other evolutionary outcomes of duplicate genes. In the classical model of divergence by *neofunctionalization*, one gene retains the ancestral activity while its paralog accumulates mutations at a higher rate and is occasionally fixed in a population by the acquisition of an adaptive function (Ohno, 1970). In contrast, the *subfunctionalization* model considers that, after gene duplication and divergence, the biological or molecular functions of the ancestor become partitioned between the paralogs. The term subfunctionalization has been used it two different evolutionary models. In the escape from adaptive conflict model (Hughes, 1994; Sikosek et al., 2012), adaptive evolution leads to a qualitative subfunctionalization of the molecular functions that tradeoff between each other in the ancestral gene. Each paralog may then evolve toward the optimization of the retained function. Alternatively, quantitative subfunctionalization occurs when neutral evolution results in complementary loss-of-function mutations between the paralogs. In this model, both duplicates become indispensable as they together provide the ancestral functional requirements (Force et al., 1999; Lynch and Force, 2000; He and Zhang, 2005b). Finally, in the *gene-dosage amplification* model, a gene pair may be retained in duplicate by the selective advantage of increasing the dosage of the ancestral function (Kondrashov et al., 2002; Kondrashov and Kondrashov, 2006; Tang and Amon, 2013).

Complete functional redundancy between the paralogs is expected immediately after gene duplication. The initial redundancy of duplicate genes can help to overcome the tradeoffs associated to the evolution of functional novelty (Conant and Wolfe, 2008; Soskine and Tawfik, 2010). The presence of duplicate genes with overlapping roles may relax the selection pressure and enable innovation (Zhang, 2003; Conant and Wolfe, 2008; Innan and Kondrashov, 2010). After divergence, certain degree of functional overlap may remain through long evolutionary times (Ihmels et al., 2007; DeLuna et al., 2008).

## Gene Duplication May Enable the Origin of Moonlighting Functions

**FIGURE 1:**
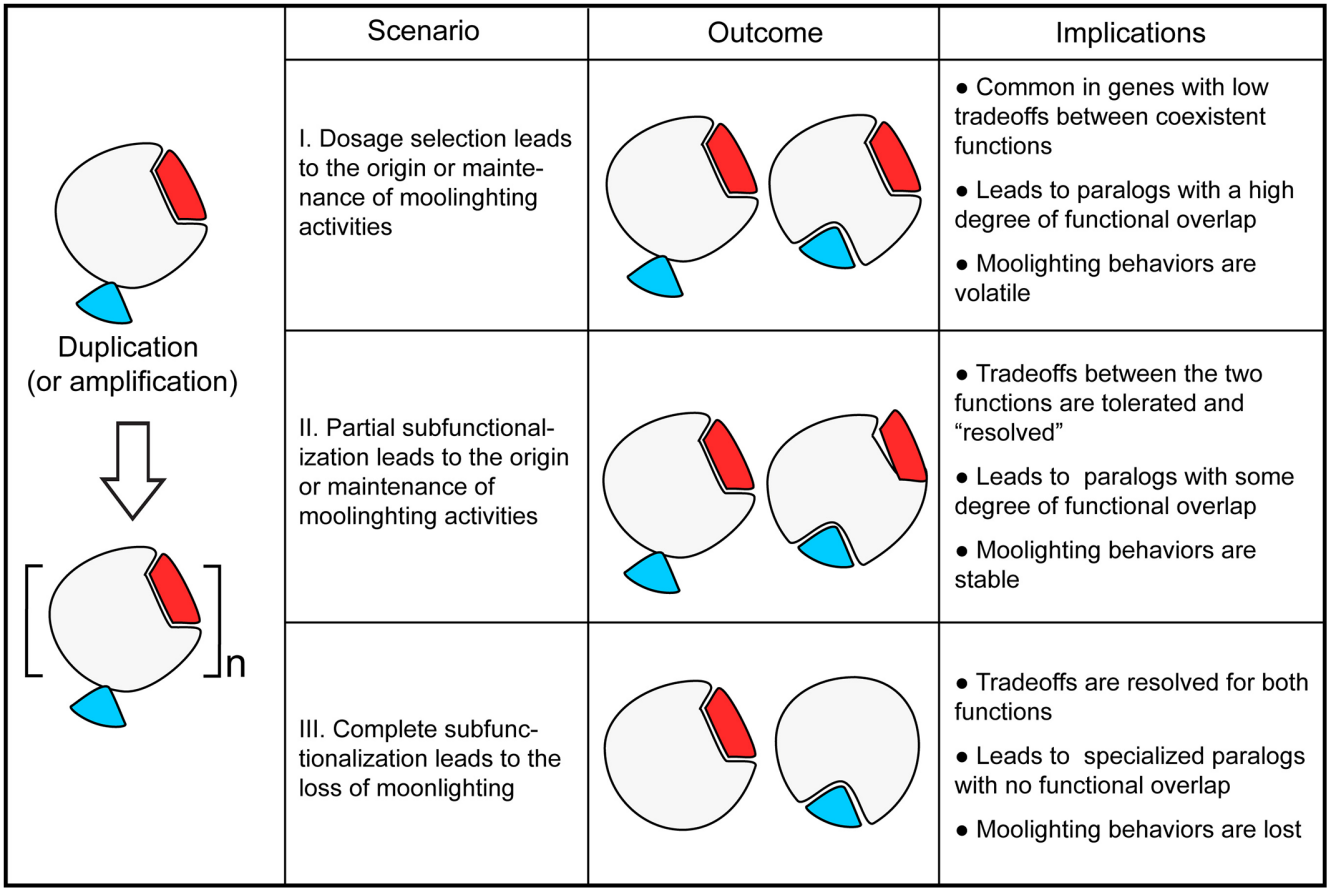
**The fate of moonlighting proteins after gene duplication.** The interplay between different mechanisms of gene evolution by duplication (including amplification) influence the origin, retention, and loss of moonlighting proteins. (I) Gene duplication may enable the origin or retention of moonlighting proteins over time by selection for dosage amplification of one or more molecular functions in the ancestor (e.g., yeast Rpl2A/Rpl2B). (II) Incomplete subfunctionalization of one or more molecular activities in the ancestor may enable the origin or retention of moonlighting behaviors as a result of neutral evolution (e.g., yeast Eno1/Eno2). In this scenario, the subfunctionalization may act on gene expression or protein activity, e.g., substrate specificity. (III) In contrast, duplication may result in the loss of moonlighting behaviors of one of the paralogs (e.g., yeast Gal1/Gal3), or of in both gene products (e.g., chicken argininosuccinate lyase/δ-crystallins) by complete specialization of their molecular activities.

With the evolutionary models of gene duplication in mind, we examined available databases of moonlighting proteins described to date (Hernandez et al., 2014; Mani et al., 2015). Many of these proteins have gone through gene duplication events. In the specific example of the budding yeast *Saccharomyces cerevisiae*, for which extensive functional data is available, over 30 such multifunctional proteins have been described. This set includes 14 genes with paralogs that originated either from the whole-genome duplication or from small-scale duplication events (**Table 1**). Therefore, it is tempting to speculate that duplication dynamics have played a role in the origins and maintenance of gene multifunctionality. In what follows, we present different scenarios of evolution of moonlighting proteins by gene duplication, starting from ancestral monofunctional or multifunctional states and driven by neutral or adaptive evolution (see **Figure 1**).

**Table 1:** Moonlighting paralogs in *Saccharomyces cerevisiae*.

Moonlighting gene	Paralog^a^	% ID^b^	Conserved moonlighting^c^	Source^d^	Function	Additional function(s)
*RPS14A*	*RPS14B*	98	Yes*	1	Component of the ribosome small subunit (40S)	Represses the expression of RPS14B
*RPL2A*	*RPL2B*	100	Yes*	1	Component of the ribosome large subunit (60S)	Regulates the accumulation of L2 mRNA
*RPS28A*	*RPS28B*	99	Yes*/**	1	Component of the ribosome small subunit (40S)	Shortens the half-life of its own mRNA
*HXK2*	*HXK1*	77	Yes (see text)	1,2	Hexokinase	Transcriptional regulator
*ENO1*	*ENO2*	95	Yes (see text)	1,2	Enolase	Required for vacuole homotypic membrane fusion and protein trafficking to the vacuole. Involved in tRNA mitochondrial targeting
*ENO2*	*ENO1*	95	Yes (see text)	1	Enolase	Required for vacuole homotypic membrane fusion and protein trafficking to the vacuole. Involved in tRNA mitochondrial targeting
*CYC1*	*CYC7*	84	No	1	Component of the mitochondrial electron-transport chain	Promotes apoptosis
*SIS2*	*VHS3*	63	Yes (see text)	1,2	Subunit of phosphopantothenoylcysteine decarboxylase (PPCDC)	Inhibitory subunit of protein phosphatase Ppz1
*VHS3*	*SIS2*	63	Yes (see text)	1,2	Subunit of phosphopantothenoylcysteine decarboxylase (PPCDC)	Inhibitory subunit of protein phosphatase Ppz1
*TSA1*	*TSA2*	86	Yes**	1,2	Peroxiredoxin peroxidase	Molecular chaperone
*TSA2*	*TSA1*	86	Yes**	1	Peroxiredoxin peroxidase	Molecular chaperone
*CCM1*	*PET309*	27	No	2	Supports bI4 maturase activity (removal of COB and COX1 fourth introns)	Key to maintain the steady-state levels of the mito-ribosome small subunit RNA
*GAL1*	*GAL3*	74	No (see text)	3	Galactokinase	Transcriptional regulator
*LYS20*	*LYS21*	92	Yes**	4	Homocitrate synthase	DNA-damage repair

*^a^NCBI blastp suite-2sequences.*

*^b^Obtained from the Fungal Orthogroups Repository (http://www.broadinstitute.org/regev/orthogroups/).*

^c^(*) Sequence conservation suggest that both copies retain the moonlighting behavoir; (**) Phenotypes of the paralog suggest that moonlighting activity is preserved.

*^d^(1) Moonprot (Mani et al., 2015); (2) MultitaskProtDB (Hernandez et al., 2014); (3) Abramczyk et al., 2012; (4) Scott and Pillus, 2010.*

Moonlighting proteins are single-gene products with more than one molecular function. Therefore, the origin of a new moonlighting role implies that a new molecular function is fixed and that the ancestral activity is maintained. As discussed above, the tradeoffs between the origin of functional novelty and the maintenance of an ancestral activity may be overcome by the presence of duplicated genes, where loss-of-function mutations in one copy may be compensated by the paralog. However, the ancestral function is expected to be fully or partially conserved under at least two scenarios whereby both gene copies are rendered essential: selection for *gene-dosage amplification* (Kondrashov and Kondrashov, 2006; Katju and Bergthorsson, 2013) or *incomplete subfunctionalization* (Force et al., 1999; Lynch and Force, 2000; Fares, 2014). We define incomplete subfunctionalization as the phenomenon where, after duplication, a certain degree of functional overlap is retained between the paralogs. Incomplete subfunctionalization may reflect both neutral drift (quantitative subfunctionalization) and adaptive evolution (qualitative subfunctionalization). Examples from yeast protein-interaction networks and human gene-expression profiles suggest that the combination of neofunctionalization and subfunctionalization is prevalent (He and Zhang, 2005b). We thus propose that gene duplication enables the acquisition of moonlighting functions in one or more paralogs when functional novelty is associated to a selective pressure to maintain ancestral functions either by dosage amplification selection or incomplete subfunctionalization.

Genes that are not fixed in duplicate might still gain multifunctional behaviors by transient duplication states. Full functional redundancy between paralogs immediately after gene duplication may enable the fixation of functional novelty, whereas the loss of the ancestral function is constricted by selection at least in one copy. Transient duplications, therefore, may facilitate the fixation of *de novo* moonlighting singletons. Although difficult to test, this hypothesis implies that single-copy multifunctional genes may be common in genomes that have gone through whole-genome duplication events or that are prone to frequent gene amplifications.

## Are Moonlighting Functions Maintained After Gene Duplication?

The scenario in which both paralogs moonlight after gene duplication seems to be quite common in yeast (**Table 1**). For instance, gene-dosage amplification selection and stoichiometric balance seem to influence the maintenance of moonlighting activities in duplicate. At least three yeast cytosolic ribosomal proteins show additional roles related to the regulation of their own RNA stability and expression (Presutti et al., 1991; Fewell and Woolford, 1999; Badis et al., 2004). Strong stoichiometric balance constraints and high gene-expression level requirements may have led to the maintenance of these essential genes in duplicate. The high level of sequence conservation in these slow-evolving paralogs suggests that their moonlighting capacities have been maintained after gene duplication.

In addition to gene-dosage amplification, subfunctionalization seems prevalent in the conservation of moonlighting behaviors after gene duplication. For example, Sis2 and Vhs3 participate in the same coenzyme-A synthesis protein complex and are negative regulators of a serine/threonine phosphatase (Ruiz et al., 2009). These paralogs have similar expression patterns but are partially specialized in their physical and genetic interactions. Meanwhile, both yeast enolases Eno1 and Eno2 stimulate vacuole fusion, regulate selective protein trafficking to the vacuole, and are involved in mitochondrial tRNA import (Decker and Wickner, 2006; Entelis et al., 2006; Gancedo and Flores, 2008). *ENO2* is an essential gene and is expressed in the presence of glucose at a 20-fold higher level than its paralog. Eno1 and Eno2 have specialized in their catalytic properties and expression patterns, while their moonlighting behavior has been maintained (McAlister and Holland, 1982; Entian et al., 1987).

Even for duplicate pairs in which only one copy has been defined moonlighting, it is common to find evidence of some degree of multifunctional behavior in its paralog. The yeast hexokinases Hxk1 and Hxk2 provide such an example. Both duplicates catalyze the phosphorylation of hexoses under different biological conditions: *HXK2* is expressed in growth under glucose, whereas *HXK1* is expressed under different carbon sources (Rodriguez et al., 2001; Flores and Gancedo, 2011; Gancedo et al., 2014). Besides phosphorylating hexoses, Hxk2 has been shown to play a role in the regulation of gene expression by directly binding to the Mig1 transcription factor. Evidence suggests that Hxk1 may have the same activity, since its overexpression complements the Δ*hxk2* gene-regulation phenotype (Rodriguez et al., 2001). Likewise, Lys20 has been shown to have a role linked to the repair of DNA damage by direct interaction with histone acetyltransferases, in addition to its homocitrate synthase activity critical for lysine biosynthesis (Scott and Pillus, 2010). Although only Lys20 was characterized as a moonlighting protein, both Lys20 and Lys21 have been detected in the nucleus in a chromatin-bound, not freely diffusible form (Chen et al., 1997). Moreover, overexpression of *LYS21* suppresses the histone-acetyltransferase mutant phenotype, but to a lesser degree than *LYS20* (Scott and Pillus, 2010). Other moonlighting proteins with overlapping, conditional activities may remain to be identified.

In the examples provided above, we assume that the paralogs originated from a moonlighting ancestor. The *GAL1*/*GAL3* pair is perhaps the only example presented in **Table 1** with conclusive evidence that one paralog has lost the multifunctional character of the ancestral moonlighting protein (Platt et al., 2000). These genes are a representative case of the escape from adaptive conflict model, whereby the duplicates have specialized: Gal1 retains the galactose-phosphorylation activity while Gal3 is a transcriptional regulator (Hittinger and Carroll, 2007). It is interesting to note that Gal1 maintains the transcriptional regulator activity under specific conditions, such as the absence of *GAL3* and during diauxic shift (Abramczyk et al., 2012). Therefore, even in the canonical example of loss of moonlighting behavior by complete subfunctionalization following gene duplication, there is evidence of partial conservation of an additional gene function.

In conclusion, many duplicates seem to preserve partially overlapping ancestral and moonlighting functions despite long evolutionary time. Dosage selection and incomplete subfunctionalization seem to be key mechanisms of functional preservation after gene duplication. Since subfunctionalization relies on the partition of ancestral properties, this mechanism is expected to play a role in the evolution of complex genes-that is, pleiotropic genes or genes with different expression patterns-or multifunctional genes (He and Zhang, 2005a).

## Mechanisms of Moonlighting Maintenance by Gene Duplication

Molecular functions of moonlighting paralogs may be conserved by selection of gene-dosage amplification. Although genes under such pressure evolve slowly (Jordan et al., 2004; Drummond et al., 2005), functional divergence between such paralogs is not unusual. For example, yeast histones are correlated in their expression partners, are highly expressed, but show specific patterns of genetic interactions (Ihmels et al., 2007). Therefore, evolution by gene duplication and dosage amplification selection may allow certain degree of specialization and resolution of molecular tradeoffs between the coexisting functions of moonlighting proteins.

Incomplete subfunctionalization also leads to functional conservation of moonlighting paralogs. Rapid gene divergence is considered to occur typically in *cis*-regulatory regions, resulting in paralogs with the same biochemical properties but different expression patterns (Li et al., 2005; Duarte et al., 2006; Gout and Lynch, 2015). Regulatory divergence may also affect cellular localization or alternative splicing. Nonetheless, the intrinsic biochemical activities may also diverge and result in differences in specificity, affinity, or direction of reaction (Hoekstra and Coyne, 2007; Xu et al., 2012). An initial fast subfunctionalization of the expression pattern after duplication may favor the retention of both ancestral and moonlighting functions. Meanwhile, specialization of the individual molecular activities may occur continuously. Therefore, incomplete subfunctionalization may also resolve molecular tradeoffs and allow the long-term maintenance of moonlighting proteins in a context of functional redundancy.

The “problem” of genetic redundancy is the apparent paradox that seemingly dispensable gene copies are retained at the long term (Nowak et al., 1997). Protein moonlighting exacerbates the problem of genetic redundancy: not only redundant genes are retained, but more than one molecular function is conserved in duplicate. It has been argued that functional redundancy between paralogs can be selected to confront environmental, genetic, or stochastic perturbations (Gu et al., 2003; Keane et al., 2014; Noman et al., 2015). Even duplicates that have noticeably diverged in regulation or molecular function can provide some degree of genetic buffering (Ihmels et al., 2007; DeLuna et al., 2008; VanderSluis et al., 2010; Diss et al., 2014). Moonlighting paralogs may therefore be maintained as a mechanism of genetic robustness.

Several duplicate moonlighting genes from yeast show mechanisms of buffering that can provide genetic robustness. For instance, Eno2, Hxk1, and Lys20/21 are up-regulated in response to deletion of their paralogs (DeLuna et al., 2010). Such *paralog responsiveness* is exposed under particular genetic or environmental contexts. Interestingly, Hxk1 and Lys21 respond to the absence of their paralogs even under conditions in which the catalytic activities are not needed (DeLuna et al., 2010), suggesting compensation between the moonlighting roles.

Other sources of redundancy and genetic robustness are also likely to play a role in the evolution of protein moonlighting. For instance, protein multifunctionality may originate even if mutations favoring the emergence of a new activity have a strong underlying tradeoff with the pre-existing function, but are tolerated at the organismic fitness level due to compensatory mechanisms (DePristo et al., 2005; Tokuriki and Tawfik, 2009a). While gene duplication is of particular importance in eukaryotic organisms, gene amplification, horizontal gene transfer, or hybridization are more recurrent in prokaryotes and could also influence the maintenance and generation of moonlighting proteins in these organisms. Indeed, an examination of examples available for *Escherichia coli* (Hernandez et al., 2014; Mani et al., 2015) suggest that about half of the characterized moonlighting proteins have at least one homologous copy in the genome.

Different scenarios of evolution of moonlighting proteins by duplication and amplification are not mutually exclusive and may coexist with previously proposed mechanisms, such as repurposing or tinkering. The outcomes are likely influenced by the structural properties of proteins and of the mutations that occur in *cis* or in *trans*. In conclusion, an interplay between the strength of the molecular tradeoffs between functions, the selection for their conservation mediated by dosage selection or rapid changes in gene expression, and genetic robustness shape the evolution of moonlighting duplicate genes for long evolutionary times.

## Future Directions

The evolutionary pathways leading to the generation, retention, and loss of moonlighting proteins remain largely unknown. The actual extent to which gene duplication has contributed to their evolution may be elucidated as we gain insights into additional examples in yeast and in other organisms. Genetic complementation with the paralogous sequences of known moonlighting proteins could shed light into the conservation and volatility of multifunctional behaviors. Given that molecular roles are usually exposed under specific cellular contexts, challenging organisms systematically with different environmental and genetic perturbations will help to identify novel examples of moonlighting proteins. In addition, analyses of sequenced genomes could provide a means to predict and annotate this emerging type of functional behavior, especially in non-conventional species that are closely related to model organisms. For instance, lineage-specific duplicated genes with particular functional profiles may be good starting point to look out for proteins with extra biological roles.

As with most studies of genes and gene functions, the focus on moonlighting proteins and their evolution necessarily touches on the more fundamental question of how we define and detect biological “function.” Approaching the problem in more comprehensive and systematic ways will ultimately impact our understanding of the evolution of both duplicate genes and moonlighting proteins.

## Conflict of Interest Statement

The authors declare that the research was conducted in the absence of any commercial or financial relationships that could be construed as a potential conflict of interest.
